# Who live longer than their age peers: individual predictors of longevity among older individuals

**DOI:** 10.1007/s40520-022-02323-5

**Published:** 2022-12-30

**Authors:** Lily Nosraty, Dorly Deeg, Jani Raitanen, Marja Jylhä

**Affiliations:** 1 Faculty of Social Sciences (Health Sciences) and Gerontology Research Center (GEREC), Tampere University, N33014 Tampere, Finland; 2 Department of Epidemiology and Data Science, and Amsterdam Public Health Research Institute, Amsterdam University Medical Centers, VU University, Amsterdam, The Netherlands; 3 Faculty of Social Sciences (Health Sciences) and Gerontology Research Center (GEREC), The UKK Institute for Health Promotion Research, Tampere University, Tampere, Finland

**Keywords:** Individual-based measure of longevity, Mortality, Relative measure, Absolute measure

## Abstract

**Background:**

There are a very few studies focusing on the individual-based survival with a long follow-up time.

**Aim:**

To identify predictors and determine their joint predictive value for longevity using individual-based outcome measures.

**Methods:**

Data were drawn from Tampere Longitudinal Study on Aging (TamELSA), a study of individuals’ age 60–89 years (*N* = 1450) with a mortality follow-up of up to 35 years. Two measures of longevity were used: the longevity difference (LD) and realized probability of dying (RPD), both of which compare each individual’s longevity with their life expectancy as derived from population life tables. Independent variables were categorized into five domains: sociodemographic, health and functioning, subjective experiences, social activities, and living conditions. Linear regression models were used in three steps: bivariate analysis for each variable, multivariate analysis based on backward elimination for each domain, and one final model.

**Results:**

The most important predictors of both outcomes were marital status, years smoked regularly, mobility, self-rated health, endocrine and metabolic diseases, respiratory diseases, and unwillingness to do things or lack of energy. The explained variance in longevity was 13.8% for LD and 14.1% for RPD. This demonstrated a large proportion of unexplained error margins for the prediction of individual longevity, even though many known predictors were used.

**Discussion and conclusions:**

Several predictors associated with longer life were found. Yet, on an individual level, it remains difficult to predict who will live longer than their age peers. The stochastic element in the process of aging and in death may affect this prediction.

## Introduction

Older populations experience increasing longevity and longer old age. At the same time, a large variation in length of life exists among individuals of the same age and sex [1]. Factors that predict survival and longevity are an ongoing area of interest for epidemiologists, gerontologists, and scholars in other fields of social and health sciences. Such factors also likely drive the future development of length of life span in the population.

Since Erdman Palmore´s [2, 3] early work, evidence has continued to accumulate on the associations of medical, biological, and other health indicators, social and psychological factors, and demographic characteristics with survival [4–7]. The importance of predictors varies according to the age and other characteristics of the population under study, the length of the follow-up, the methods of analysis, the number and details of the variables, and the combinations of variables included in the final models of prediction. Overall, research indicates that the most significant predictors of mortality are multi-morbidity [8], cardiovascular disease [9], functional ability [10–12], self-rated health [1, 13], and cognitive ability [14]. Male sex and lower socioeconomic status are often mentioned as well [15], but the role of these distal indicators varies depending on the coverage and quality of the more proximal indicators. The relative predictive power of mortality risk factors seems to decline toward extremely high age [1, 16], possibly due to a ceiling effect, that is, very high baseline mortality [17], and because with increasing age death is the end result of several contributing factors and therefore “stochastic” by nature [1].

Most studies are aimed at identifying the most important individual predictors, and little is known on to what extent the variance in the long term of mortality/survival can be predicted by combinations of individual characteristics measured [18]. In this study, we investigated the individual and combined predictors of mortality/survival during a long follow-up time in an older population. By doing that, our aim is to investigate the predictors of longevity of an individual advantage compared to the life expectancy of the respective cohort.

We used individual measures of longevity and related them to actuarial life expectancies of each age cohort in the study. Different from the Cox regression model, the most frequently used method in survival/mortality studies, this method allows the calculation of the variance in mortality/survival explained by the selected variables. The method is also better suited for the study of longevity in samples with a wide age range [19]. Deeg et al.[18] and Rutherford et al. [20] suggest that to improve accuracy in longevity predictions, individual-based measures of survival time will be more sensitive to capturing heterogeneity. Further, to maximize predictive value, it is necessary to have a large enough non-selective sample and a long enough follow-up during which the majority of the sample has died; to know the exact length of survival time; and to have a wide range of potential predictors.

In this study, the aim is (1) to identify predictors of longevity in a population-based sample of people aged 60 + at baseline, and (2) to establish the joint predictive value of the combined set of predictors to investigate, what proportion of variance in survival/mortality can be explained by this combination. Our data include potential predictors from various domains describing health, functioning, social conditions and social activity measured at the baseline. We have available a relatively large population-based sample and an exceptionally long follow-up period up to 35 years. As a cross-validation, we use two different longevity measures that are based on comparisons of each individual’s survival with age- and sex-specific survival data drawn from population tables.

## Methods

### Data sources

The data for our study came from the Tampere Longitudinal Study on Aging (TamELSA) in Finland [21]. TamELSA was undertaken as part of the Eleven Countries Study initiated by the WHO European Office in 1979 [22, 23]. The study area, Tampere, is the largest mainland city of Finland, with population of 165 000 in 1979 (ca 244 000 in 2021). At the time of the data collection, the percentage of individuals aged 60 and older was slightly higher (17.0%) than in the country as a whole (16.2%). The ethnic and cultural backgrounds were largely similar and homogeneous. The data were collected using structured questionnaires in face-to-face interviews. The 1979 sample consisted of 1,059 individuals aged 60–89 years, 49.8% male, with an 81% response rate. In 1989, a new cohort of 395 people aged 60–69 years (52% male with a 76% response rate) was added [21].

The samples were combined to increase the number of the participants and subsequently the statistical power; the total sample comprised 1,454 individuals Just over half, 50.8% of them were men and 51% were younger individuals aged 60–69 years.

### Measures of longevity

Dates of death were obtained from the Finnish Population Register. Vital status was ascertained up to 1 January 2015. Four participants were excluded from the analysis since the information about their vital status were not available (1450 individuals). By the end of study, 1338 participants died and 7.7% of participants were still alive. The maximum length of follow-up was 35 years.

We used two individual measures of longevity calculated according to each participant’s age and sex and based on the population life table. These measures allow the use of linear regression to provide the variance explained by potential predictors of longevity.

The realized probability of dying (RPD) is a relative measure of longevity that is based on comparison of the survival time of each individual of a specific age and sex with the survival time of his/her peers in the total population [18, 19, 24]. An individual’s time of death is compared with the cohort survival curve for the general population of the same age and sex, calculated from the study’s baseline onwards. An individual’s RPD is expressed as the proportion of the pertinent cohort still alive at the time of death of the individual. Values are between 0 and 1, with higher values indicating shorter survival. For example, if at the time of death of an individual 90% of his/her cohort is still alive, the value of an individual’s RPD is 0.90.

For those ca 8% who were still alive at the end of the study, the RPD value was imputed by multiplying the probability of survival in 2014 by 0.50. This multiplying factor is the expected value of RPD in the population alive at any moment in time (median population survival time).

The longevity difference (LD) is an absolute measure of longevity that is calculated as the difference between the number of years an individual survived after baseline and the actuarial life expectancy (LE) in his/her year of death, based on their sex and age [25]. Higher values indicate longer survival. For example, if a male individual at the age of 65 in the year 1979 died 15 years later, the life expectancy for 65 years old male being 12.67 at the year of his death, LD for him will be 2.33.

If a participant was alive at the end of the follow-up, the LD for this subject was estimated as the sum of the number of years from baseline and the LE based on the subject’s age and sex in 2014. The LD ranged between − 19.4 and 18.0 years.

The distribution of LD was near-normal, while the distribution of RPD was near-uniform. To normalize the distribution of RPD, log-transformation was used with the formula log [RPD/(1—RPD)]. This variable (LRPD) was used in the analyses.

The life tables for the Finnish population were obtained from the Human Mortality Database (HMD) [26] for the years 1979–1985, and from Official Statistics Finland [27] (OSF) for the period from 1986 to the end of 2014. HMD was used as data for those years were not available as electronic files from the OSC; yet all the data at HMD also originally comes from the OSC. for the period from 1986 to the end of 2014.

### Predictors of longevity

Potential predictors were categorized into five domains (Table 1).

**Table 1:** Percentages of the categories of the variables, and associations of potential individual predictors with longevity difference (LD) and log-transformed realized probability of dying (LRPD); linear regressions, unstandardized coefficients (B) and p values

Domain	Variable and categories			%	LD		LRPD	
					B	*p* value	B	*p* value
Domain 1: Sociodemographic	Sex: (1) male, (2) female		Male	50.70	0.45	0.16	− 0.01	0.30
	Age (in the interview year)		Mean	71.20	0.03	0.16	− 0.02	< 0.001
	Marital status	Married		51.10	Ref			
		Never married		12.30	−0.70	0.15	0.16	0.24
		Widowed		28.90	− 0.23	0.51	− 0.06	0.56
		Divorced		7.20	− 1.42	0.02	0.35	0.04
	Years in full-time education (median)		Median	6.00	0.13	< 0.001	− 0.02	0.07
	Having children		No	20.60	0.73	0.07	− 0.12	0.26
Domain 2: Health and functioning	ADL (five activities)^a^		Mode	15.00	0.31	< 0.001	− 0.10	< 0.001
	Mobility^b^		Mode	15.00	0.27	< 0.001	− 0.08	< 0.001
	Demanding activities^c^		Mode	12.00	0.29	< 0.001	− 0.08	< 0.001
	Self-rated health		Poor	6.50	1.28	< 0.001	− 0.34	< 0.001
			Fairly poor	17.70				
			Average	36.90				
			Fairly good	30.20				
			Very good	8.70				
	Years smoked regularly		Mean	29.60	− 0.08	< 0.001	0.02	< 0.001
	Regular physical exercise		Yes	58.10	− 1.13	< 0.001	0.24	0.01
	Feeling depressiveness		Yes, nearly continuously	4.40	0.62	< 0.001	− 0.16	< 0.001
			Yes, often	5.20				
			Yes, occasionally	19.40				
			No	70.90				
	Worsening of memory		Yes, nearly continuously	14.20	0.33	0.03	− 0.05	0.21
			Yes, often	10.90				
			Yes, occasionally	26.40				
			No	48.50				
	Neoplasm		Yes	3.20	− 3.23	< 0.001	0.92	< 0.001
	Endocrine and metabolic disorders		Yes	13.80	− 1.99	< 0.001	0.52	< 0.001
	Infectious diseases		Yes	2.40	− 1.35	0.20	0.36	0.20
	Diseases of blood		Yes	1.70	− 0.59	0.64	− 0.06	0.86
	Mental disorder		Yes	3.50	− 0.68	0.44	0.32	0.17
	Nervous system diseases		Yes	23.20	− 0.34	0.36	0.05	0.65
	Hypertensive diseases		Yes	33.40	− 1.23	< 0.001	0.33	< 0.001
	Cardiac ischemic diseases		Yes	12.70	− 1.85	< 0.001	0.49	< 0.001
	Circulatory diseases		Yes	33.10	− 1.01	< 0.001	0.25	0.01
	Respiratory diseases		Yes	12.30	− 2.20	< 0.001	0.58	< 0.001
	Digestive diseases		Yes	14.50	− 0.30	0.51	0.15	0.22
	Genito-urinary diseases		Yes	8.00	− 0.12	0.84	0.08	0.62
	Skin diseases		Yes	3.80	− 0.11	0.90	0.09	0.68
	Musculoskeletal diseases		Yes	38.20	0.38	0.25	− 0.07	0.46
Domain 3: Subjective experiences	Pain in the joints or back trouble		Yes, nearly continuously	23.30	0.41	< 0.001	− 0.13	< 0.001
			Yes, often	15.80				
			Yes, occasionally	23.40				
			No	37.50				
	Satisfied with present life		Very unsatisfied	2.50	0.55	< 0.001	− 0.13	0.01
			Unsatisfied	3.20				
			Reasonably satisfied	20.90				
			Satisfied	43.40				
			Very satisfied	30.10				
	Satisfied with human relationships		Unsatisfied	9.10	0.43	< 0.001	− 0.10	0.01
			Very satisfied	90.90				
	Tired of life		Often	5.00	1.15	< 0.001	− 0.28	< 0.001
			Sometimes	24.80				
			Never	70.20				
	Unwillingness to do things or lack of energy		No or occasionally	82.50	− 1.00	< 0.001	0.26	< 0.001
			Yes often	7.40				
			Yes, occasionally	10.10				
Domain	Variable			%	LD		LRPD	
					B	*p* value	B	*p* value
Domain 3: Subjective experiences	Satisfaction with financial situation		Poor	6.80	0.85	0.01	− 0.20	0.02
			Satisfactory	66.00				
			Good	27.20				
	Feeling lonely		Often	8.90	0.69	0.01	− 0.14	0.05
			Sometimes	24.10				
			Never	67.00				
	Feeling forgotten		Often	3.40	0.78	0.02	− 0.19	0.04
			sometimes	15.60				
			Never	81.00				
	Feeling unnecessary		Often	9.70	0.91	< 0.001	− 0.19	0.01
			Sometimes	23.70				
			Never	66.60				
	Tiredness or feelings of faintness		Yes, nearly continuously	11.60	− 0.72	< 0.001	0.17	< 0.001
			Yes often	9.60				
			No or occasionally	78.80				
Domain 4: Social activities	Social activities ͩ		Median	12.00	0.09	< 0.001	− 0.02	< 0.001
	Social contacts (last paid visit)		More than half year ago	7.20	0.32	< 0.001	− 0.07	< 0.001
			About half a year ago	5.30				
			About a month ago	12.30				
			About 2 weeks ago	14.70				
			About a week ago	17.90				
			Some days ago	24.30				
			Today or yesterday	18.50				
	Social contacts (last received visit)		More than half year ago	1.20	− 0.03	0.78	0.03	0.39
			About half a year ago	1.90				
			About a month ago	6.80				
			About 2 weeks ago	8.10				
			About a week ago	15.90				
			Some days ago	27.70				
			Today or yesterday	38.30				
	Having good friends		Yes	81.70	− 0.69	0.12	0.11	0.34
	Helping to raise grandchildren		Yes	28.30	− 1.16	< 0.001	0.23	0.02
Domain 5: Living condition	Telephone at home		Yes	77.70	− 0.75	0.10	0.11	0.35
	Washing machine at home		Yes	58.00	− 0.28	0.42	− 0.02	0.83
	Fridge at home		Yes	90.10	− 0.01	0.76	− 0.21	0.46
	Freezer at home		Yes	37.40	− 0.15	0.66	− 0.10	0.29
	Use of a car		Yes	77.70	0.71	< 0.001	0.03	0.78
	Being alone		Yes	48.30	0.57	0.09	− 0.04	0.70

^a^ADL (activities of daily living): five activities as getting in and

out of bed, washing and bathing oneself, using the lavatory, dressing and undressing, and feeding oneself

^b^Mobility: able to move outdoors, walking between rooms, using stairs, walking at least 400 m, and carrying a heavy bag of 5 kg for 100 m

^c^Demanding activities: able to cut toenails, cooking, light housework and heavy housework

^d^Social activities: number of engagements in social activities: 1) family ceremonies, parties,…, 2) theater, movie,…3) visits to clubs,…, 4) library, 5) sport competitions, either watching or participating, 6) religious service, 7) traveling in foreign countries, or 8) traveling in home country

All diseases were categorized as 0: no and 1: yes

Sociodemographic characteristics included age, sex, marital status, and years in full-time education.

Health and functioning were described by the following variables: Self-reported diseases were coded into 17 categories according to the Finnish Edition of the International Classification of Diseases 1975 Revision (ICD-9), with the exception that cardiovascular diseases were divided into three subgroups: hypertonia, ischemic heart diseases, and other cardiovascular diseases.

Activities of daily living (ADL), mobility, and demanding activities were measured based on questions with four response options: (a) can do without difficulty, (b) can do with difficulty but without help, (c) can do only with help, and (d) cannot do. ADL was measured as ability to get in and out of bed, wash and bath, use the lavatory, dress and undress, and feed oneself. It was scored on a scale of 0–15, a lower score indicating poorer ADL functioning. Mobility was measured as ability to (a) move outdoors, (b) walk between rooms, (c) use stairs, (d) walk at least 400 m, and (e) carry a heavy bag of 5 kg for 100 m. It was scored on a scale of 0–15. Assessments of demanding activities comprised the ability to cut toenails, cook, do light housework, and do heavy housework. The variable scores ranged from 0 to 12.

Self-rated health was reported in five categories from very good to poor and was scored as (0) poor, (1) fairly poor, (2) average, (3) fairly good, (4) very good.

Worsening of memory and low spirits or depression were inquired using the preset response options (a) no, (b) yes, occasionally, (c) yes, often, and (d) yes, nearly continuously. Each predictor was scored on a scale of 0–3.

Regular physical exercise was coded as yes or no.

Number of years of regular smoking was considered as a continuous variable (0 as never-smoker).

#### Social activity

Social participation was measured by the number of engagements in social activities during the past 12 months. The activities specified were (a) family ceremonies, parties, weddings, and funerals, (b) theater shows, movies, concerts, and art exhibitions, (c) visits to clubs or societies, (d) library, (e) sport competitions, either watching or taking part, (f) religious service, g) traveling to foreign countries or in home country. Social participation was scored on a scale of 0–51.

Social contacts were measured as time since last visit received and last visit paid. Both had seven options from today or yesterday to more than six months ago, and both were scored from 0 to 6.

Helping to raise grandchildren and having good friends were both coded as yes or no.

#### Subjective experiences

Questions concerning unwillingness to do things or lack of energy and tiredness or feeling of faintness had four response options from no to yes; occasionally, often, and continuously, and were scored on a scale of 0–3.

Feeling forgotten, feeling unnecessary, feeling tired of life, and feeling lonely were coded as (0) often, (1) sometimes, (2) never. These variables were on a scale from 0 to 2.

Satisfaction with present life was coded as (4) very satisfied, (3) satisfied, (2) reasonably satisfied (1), unsatisfied, and (0) very unsatisfied. The variable was scored on a scale of 0–4.

Satisfaction with human relationships was coded as satisfied (1) and unsatisfied (0). The variable was scored as 0–1. Satisfaction with personal financial situation was coded as poor (0), satisfactory (1), and good (2).

Pain in joints or back trouble were coded as (0) yes, nearly continuously, (1) yes, often, 2) yes, occasionally, and (3) no, and scored 0–3.

#### Living conditions

Living conditions included questions on having a washing machine, telephone, freezer, and refrigerator, and having the use of a car; responses were coded as yes or no.

Being alone was coded as often, rarely, or never, ranging from 0 to 2.

### Analysis

Age, number of years smoked regularly, years in full-time education, and the score of social activity were available as continuous variables. Nominal and ordinal variables were treated as continuous variables, with the exception of sex and marital status.

An effect size calculation with a power of 0.8 was conducted. Given the sample size of 1450, the study was sufficiently powered for an effect size that was 0.07 or larger to be meaningful. The analyses for both outcome variables were conducted in three steps to maximize the variance explained and to minimize the number of variables included in the final model.

First, bivariate associations of each potential predictor with the outcomes were analyzed (Table 1) and variables were identified as potential predictors if they presented a significant association (*p* ≤ 0.20) with LD or LRPD, respectively.

Second, the variables that showed a significant association with the outcome, 39 variables for LD and 32 variables for LRPD, were included in multivariate analysis within each domain. Backward linear regression was performed for LD and LRPD separately to determine the variance explained by each domain. Predictors from each domain that were significant at *p* ≤ 0.20 were retained to the next step.

Third, full linear regression models were performed with 14 variables for LD and 18 variables for LRPD, respectively, to determine which variables retained predictive power when predictors from other domains were included. At this step, a backward linear regression was performed for both outcome variables to identify the most parsimonious models.

Multicollinearity was not detected when calculating the tolerance and variance inflation factor (VIF) for each variable in both final models (tolerances were > 0.1 and VIFs < 10). The analyses were conducted using SPSS 25.

## Results

LD and RPD were found to be strongly negatively correlated (Pearson correlation *r* = − 0.96). The mean values of LD, RPD, and LRPD were 0.42, 0.44, and − 0.39, respectively. Comparison of these values with their theoretical means of 0.50 (RPD) and 0.00 (LRPD and LD) implies that the sample is slightly healthier than the general Finnish population in these age groups.

Most individual variables were associated with one or both longevity measures, the notable exceptions being some disease categories and most variables describing living conditions (Table 1). Although both LD and LRPD were defined based on age and sex, both were significantly associated with age, and LRPD also with sex in the first step.

The variance explained by domain (Table 2) ranged from 0.7% (living conditions) to 9% health and functioning) for LD and from 1.7% (social activity) to 9.1% (health and functioning) for LRPD. No variable in the domain of living conditions was significantly associated with LRPD and therefore this domain was not included in the multivariate analysis.

**Table 2:** Associations of potential predictors with longevity differences (LD) and log-transformed realized probability of dying (LRPD) by domain and variance explained by each domain

Domains and Variance explained by domain	Potential predictor		Model with all independent variables from each domain			Model after backward elimination			Model with all independent variables from each domain			Model after backward elimination		
			B	SE (B)	*p* value	B	SE (B)	*p* value	B	SE (B)	*p* value	B	SE (B)	*p* value
Domain 1: sociodemographic LD-*R*^2^ = 0.023 LRPD-*R*^2^ = 0.021	Sex		1.12	0.35	< 0.001	1.13	0.35	< 0.001						
	Age (in the interview year)		0.08	0.02	< 0.001	0.08	0.02	< 0.001	− 0.03	0.01	< 0.001	− 0.03	0.01	< 0.001
	Marital Status	Never married	1.49	0.65	0.02	− 1.81	0.56	< 0.001	0.29	0.14	0.05	0.24	0.14	0.08
		Widowed	− 1.15	0.43	0.01	− 1.18	0.43	0.01	0.12	0.11	0.26			
		Divorced	− 1.76	0.64	< 0.001	− 1.82	0.64	< 0.001	0.28	0.17	0.10			
	Years of full-time education		0.18	0.05	< 0.001	0.18	0.05	< 0.001	− 0.04	0.01	0.01	− 0.05	0.01	< 0.001
	Having children		0.57	0.48	0.24									
Domain 2: health and functioning LD-*R*^2^ = 0.090 LRPD-*R*^2^ = 0.091	ADL (five activities)		− 0.12	0.22	0.58				0.02	0.06	0.71			
	Mobility		0.23	0.15	0.12	0.18	0.09	0.04	− 0.06	0.04	0.13	− 0.05	0.02	0.03
	Demanding activities		− 0.02	0.14	0.89				0.01	0.04	0.83			
	Self-rated health		0.70	0.30	0.02	0.84	0.27	< 0.001	− 0.22	0.08	< 0.001	− 0.25	0.07	< 0.001
	Years smoked regularly		− 0.06	0.02	< 0.001	− 0.06	0.02	< 0.001	0.01	0.00	< 0.001	0.01	0.00	< 0.001
	Regular physical exercise		− 0.60	0.57	0.29				0.17	0.15	0.25			
	Feeling depressiveness		0.20	0.35	0.56				− 0.08	0.09	0.35			
	Worsening memory		− 0.46	0.25	0.07	− 0.41	0.24	0.09	0.16	0.06	0.01			
	Neoplasm		− 2.27	1.54	0.14				0.69	0.39	0.08	0.15	0.06	0.02
	Endocrine and metabolic disorders		− 2.01	0.81	0.01	− 1.99	0.80	0.01	0.39	0.21	0.06	0.73	0.39	0.06
	Infectious diseases		0.15	1.76	0.93				0.12	0.45	0.79			
	Mental disorders								− 0.71	0.41	0.08			
	Hypertensive diseases		− 0.13	0.66	0.84				0.07	0.17	0.68	0.40	0.20	0.05
	Cardiac ischemic diseases		− 0.62	0.86	0.47				0.15	0.22	0.51			
	Circulatory diseases		0.52	0.56	0.36				− 0.11	0.14	0.43			
	Respiratory diseases		− 1.37	0.70	0.05	− 1.34	0.69	0.51	0.33	0.18	0.07	0.36	0.18	0.04
Domain 3: subjective experience LD-*R*^2^ = 0.032 LRPD-*R*^2^ = 0.029	Pain in the joints or back trouble		0.12	0.15	0.43				− 0.06	0.04	0.11	− 0.07	0.04	0.08
	Satisfied with present life		− 0.02	0.21	0.94				− 0.13	0.06	0.99			
	Satisfied with human relationships		0.34	0.15	0.02	0.37	0.14	0.01	− 0.08	0.04	0.05	− 0.09	0.04	0.03
	Tired of life		0.32	0.36	0.38				− 0.09	0.10	0.36			
	Unwillingness to do things/lack of energy		− 0.81	0.23	< 0.001	− 0.95	0.17	< 0.001	0.24	0.06	< 0.001	0.24	0.05	< 0.001
	Satisfied with financial situation		0.52	0.32	0.11	0.57	0.31	0.06	− 0.11	0.09	0.21			
	Feeling lonely		0.07	0.32	0.83				0.02	0.09	0.81			
	Feeling forgotten		− 0.12	0.42	0.78				− 0.01	0.11	0.97			
	Feeling unnecessary		0.20	0.32	0.54				0.00	0.09	0.98			
	Tiredness or feelings of faintness		0.02	0.22	0.93				− 0.03	0.06	0.61			
Domain 4: social activities LD-*R*^2^ = 0.023 LRPD-*R*^2^ = 0.017	Social activities		0.08	0.02	< 0.001	0.08	0.02	< 0.001	− 0.02	0.01	< 0.001	− 0.02	0.00	< 0.001
	Social contacts (Last paid visit)		0.13	0.10	0.18				− 0.03	0.03	0.25			
	Having good friends		0.15	0.47	0.76									
	Helping to raise grandchildren		− 0.74	0.37	0.05	− 0.81	0.37	0.03	0.13	0.10	0.19			
Domain 5: living Conditions LD-*R*^2^ = 0.007	Having telephone at home		− 0.29	0.48	0.54									
	Use of a car		0.59	0.20	< 0.00	0.66	0.19	< 0.001						
	Being alone		0.32	0.36	0.38									

In the full linear regression model (Table 3), age, marital status, mobility, self-rated health, years smoked regularly, endocrine and metabolic diseases, respiratory diseases, and unwillingness to do things or lack of energy were included as predictors of both outcomes. For LD, satisfaction with personal financial situation and having the use of a car and for LRPD, neoplasms and social activities also remained in the final model. The total variance explained (*R*^2^) was 13.8% for LD and 14.1% for LRPD.

**Table 3:** Final models of predictors of longevity differences (LD) and log-transformed realized probability of dying (LRPD)

Predictors		Model with all independent variables			Model after backward elimination			Model with all independent variables			Model after backward elimination		
		B	SE(B)	*p *value	B	SE(B)	*p *value	B	SE(B)	*p *value	B	SE(B)	*p* value
Sex		− 0.25	0.73	0.74									
Age (in the interview year)		0.16	0.04	< 0.001	0.17	0.04	< 0.001	− 0.05	0.01	< 0.001	− 0.05	0.01	< 0.001
Marital status	Never married	− 1.46	1.06	0.17	− 1.76	1.03	0.09	0.52	0.26	0.04	0.48	0.25	0.06
	Widowed	− 1.08	0.69	0.12	− 1.14	0.66	0.08	0.35	0.16	0.03	0.32	0.16	0.05
	Divorced	− 1.58	0.90	0.08	− 1.82	0.86	0.03	0.33	0.22	0.13			
Years of full-time education		0.00	0.08	0.98				0.00	0.02	0.98			
Mobility		0.20	0.09	0.03	0.22	0.09	0.02	− 0.06	0.02	0.01	− 0.06	0.02	0.01
Self-rated health		0.49	0.29	0.09	0.52	0.29	0.07	− 0.12	0.08	0.12	− 0.13	0.07	0.08
Years smoked regularly		− 0.06	0.02	< 0.001	− 0.07	0.02	< 0.001	0.01	0.00	< 0.001	0.01	0.00	< 0.001
Worsening of memory		− 0.21	0.25	0.39									
Neoplasm								0.82	0.38	0.03	0.76	0.38	0.05
Endocrine and metabolic disorders		− 1.80	0.78	0.02	− 1.88	0.78	0.02	0.40	0.20	0.04	0.44	0.20	0.03
Respiratory diseases		− 1.20	0.68	0.08	− 1.19	0.67	0.08	0.31	0.17	0.08	0.30	0.17	0.08
Hypertensive diseases								0.10	0.13	0.45			
Pain in the joints or back trouble								0.02	0.05	0.78			
Satisfied with human relationships		0.22	0.23	0.34				− 0.01	0.06	0.83			
Satisfied with financial situation		− 0.84	0.46	0.07	− 0.75	0.45	0.10						
Unwillingness to do things/lack of energy		− 0.47	0.27	0.08	− 0.49	0.27	0.07	0.13	0.07	0.07	0.13	0.07	0.06
Social activities		0.04	0.03	0.18				− 0.01	0.01	0.10	− 0.01	0.01	0.07
Helping to raise grandchildren		− 0.62	0.55	0.26									
Use of a car		0.63	0.30	0.04	0.76	0.29	0.01						

Total variance explained is 13.8% for LD and 14.1% for LRPD

## Discussion

In this study, we asked, whether it is possible to predict who will live longer than their age peers. Numerous earlier studies have identified factors associated with mortality/survival, but almost all of them have focused on strength of individual risk factors in the sample studied. Our approach is different; we focus on individual longevity advantage of individuals compared to actuarial life expectancies, and aim at establishing the joint predictive value of a broad set of measures. to maximize the validity of our findings, we used both the longevity difference (LD) and the realized probability of dying (RPD) as outcome measures.

Our findings showed that many of the health and social indicators used were able to identify increased likelihood of longer or shorter longevity several decades ahead. Yet our final models could explain only less than 15% of the variance in longevity.

With a couple of exceptions, the individual indicators that remained significant in the final multivariate models were the same for both outcomes. Endocrine and metabolic diseases (most importantly diabetes), respiratory diseases, and neoplasms are known to be strong predictors of mortality [28, 29]. Self-rated health was retained with borderline significance in both models, which is consistent with several earlier studies with shorter and longer follow-ups [30, 31]. It was also a predictor of LD in the early studies by Palmore [2, 25]. Smoking and other than married marital status are also well-known predictors of mortality. A novel predictor, unwillingness to do things or lack of energy likely implies depressive feelings. Having the use of a car, another novel predictor of longevity, likely reflects both functional ability and, for older individuals in the 1970s and 1980s, socioeconomic position as well.

It is notable that several indicators of sociodemographic position, subjective experiences, and social activity, which as individual variables were strongly associated with length of live, lost their significance in multivariate models. These factors can be understood as distal predictors of mortality that impact the likelihood of mortality through their associations with health, disease and functioning. When measures describing them were included in the models, the independent association of these distal predictors disappeared.

Although both our longevity measures were based on individuals’ age and sex, which should eliminate their complicating effects at the entrance of the study [2, 18, 25], age was retained in both models. Deeg et al.[18, 19] explained a similar unexpected finding as the result of the way of imputing RPD and LD for individuals alive at the end of the follow-up. However, in the current study, only about 8% of the participants were alive at the end of the follow-up. The persistent positive association of age with LD and the negative association of age with LRPD likely reflect a bias toward an increasingly healthier selection of the initial sample at higher ages. Maintaining age in the final multivariate prediction models should eliminate such bias, however.

In spite of the wide range of measures representing different domains of life and the significant associations of several individual indicators with mortality, the total predictive value of the final models was rather low, 13.8% for LD and 14.1% for RPD. The variance of longevity remains mostly unexplained. Similar results have been reported in the few other studies using the same outcomes. For example, Deeg et al. [18] were able to explain only 25% of the variance of longevity in their 24-year follow-up study using measures from several domains of life. The low predictive values may be due to changes in health status after the baseline measurements. It is known that major chronic diseases that also are leading causes of death, such as cardiovascular diseases, cancer and dementia, are highly age-dependent and their incidence increases steeply with advancing age. These conditions, again, are known to be associated with factors measured in our study such as education, a significant predictor as an individual variable and indirectly reflected in the final model by satisfaction with financial situation and having the use of a car, and years of smoking. Functional disability, a known predictor of mortality, is also strongly associated with age and increases in frequency with every added year of life even at very old age [32]. Therefore, the major conditions predicting and leading to death were likely to appear during our very long follow-up but only after the baseline measurements. Regardless, the fact that we needed to estimate future longevity for only 8% of our sample who were still alive at the end of the follow-up, is a strong aspect of our study.

In this study, we investigated predictors of individual longevity using an exceptionally long follow-up of up to 35 years. We used a population-based sample, and almost all participants died by the end of the follow-up. We had access to reliable information on dates of death. One strength of our study was that we had access to a wide range of baseline variables that represented different domains, describing living conditions, socioeconomic position, social activity, subjective experiences, and health and functioning. Yet our models were able to explain only a small proportion of the probability of dying. Could we have achieved greater predictive value with better, particularly biological variables? In their 24-year follow-up, Deeg et al. [18] had information on ApoE gene and eight commonly used blood measurements, including creatinine, CRP and serum albumin. Together, these factors added only 3.7% to the total predictive value of the model. It is plausible to conclude that with a follow-up period of several decades, it is difficult to predict individual survival time regardless of which baseline factors are considered, as social circumstances change with time, health conditions and their severity increase, and furthermore there is a stochastic element in the process of aging and in death[11, 33].

## Conclusion

There are two different messages from our study. The regular, robust associations with factors, such as social position, functional status, and smoking, confirm earlier findings in that tackling inequality and promoting healthy lifestyles are likely to increase longevity in the population. But who lives longer than their age peers and exactly how long will individuals live—that is much more difficult to predict.
